# A Comprehensive Examination of Vegan Lifestyle in Italy

**DOI:** 10.3390/nu16010086

**Published:** 2023-12-26

**Authors:** Annachiara Stenico, Diana Zarantonello, Fabio Vittadello, Michael Kob

**Affiliations:** 1 College of Health Care-Professions “Claudiana”, 39100 Bolzano, Italy; annachiarastenico@gmail.com; 2 Department of Nephrology, S. Chiara Hospital, APSS, 38122 Trento, Italy; diana.zarantonello@apss.tn.it; 3 Explora—Research and Statistical Analysis, 350100 Padova, Italy; fabio.vittadello@centroexplora.it; 4 Division of Clinical Nutrition, Hospital of Bolzano (SABES-ASDAA), Teaching Hospital of Paracelsus Medical University (PMU), 39100 Bolzano, Italy

**Keywords:** plant-based diet, vegan diet, healthy habits and eating trends, dietary patterns

## Abstract

The popularity of veganism and plant-based diets is rapidly increasing worldwide, including in Italy, where more individuals and families are adopting this lifestyle. However, medical and health professionals often lack the necessary knowledge and are skeptical about this diet despite the scientific evidence. It is important for them to provide support and expertise to those following this diet. The survey evaluated various aspects of the lifestyle of Italian vegans living in Italy and abroad, including food frequency, vitamin and mineral supplementation, relationship with medical and health professionals, and perceived difficulties in daily life. The emphasis was on potentially critical aspects for those following this dietary choice. A cross-sectional survey was conducted in Italy between March and April 2022. A questionnaire was distributed through social media platforms such as Instagram, Facebook, and Telegram, and 2180 Italian adults who follow a vegan diet completed it. The survey found that most of the vegan population surveyed were female, showed a greater sensitivity to ethical issues, were aware of the need for vitamin B12 supplementation, and followed healthy-eating guidelines. It is evident that despite the increasing popularity of plant-based diets, many medical and health professionals remain cautious and hesitant to recommend them.

## 1. Introduction

A vegan diet is characterized by the complete exclusion of animal products, including meat, fish, dairy, eggs, and honey. In recent years, there has been a significant increase in the popularity of plant-based diets among Western populations. In the UK, 8% of people claimed to follow a plant-based diet in 2021 [1], while in the US and Australia, the figures were 1.5% in 2023 [2] and 2% in 2020 [3], respectively.

The Eurispes survey reports that the percentage of Italians following a vegan diet will increase from 1.4% in 2022 to 2.4% in 2023 [4]. Respondents cited a broader philosophy of life as their main motivation for adopting a vegan or vegetarian diet, followed by health (mental and physical wellbeing), ethics, and respect for animals. In fourth, fifth, and sixth place, respectively, additional reasons for adopting a vegetarian diet were environmental protection, experimentation with new ways of eating, and the belief in sacrificing quantity for quality by eating less but better [5]. This increase may be due to a growing concern for environmental sustainability, increased popularization of animal welfare issues, and heightened sensitivity towards personal health, as demonstrated in other populations [6].

The scientific literature confirms that a plant-based diet, when supplemented with adequate vitamin B12 and attention to critical nutrients such as protein, omega-3, iron, calcium, zinc, iodine, and vitamin D, promotes health and is associated with a lower risk of all-cause mortality. Additionally, it has been shown to be effective in treating, halting, and reversing some major diseases, including type 2 diabetes, cardiovascular disease, and cancer incidence [7,8,9]. The Academy of Nutrition and Dietetics (AND) states that plant-based diets, whether vegetarian or vegan, can be healthy, nutritionally adequate, and appropriate for individuals at any stage of life, provided they are properly planned and supplemented [10].

From an environmental perspective, a plant-based diet is considered the most effective dietary model to date. This is because emissions of pollutants, as well as land and water use, are closely related to diet [11]. A vegan diet has a favorable impact on many ecological indicators and is compatible with the ‘diet for planetary health’ recommended by the EAT-Lancet Commission. This diet has the potential to address the environmental crisis, feed the entire world population, and prevent chronic diseases [12].

Despite the evidence and confirmation, some medical professionals still hold the opinion that a vegan diet is nutritionally deficient and may not be suitable for certain patient groups, particularly pregnant and lactating women and children. A study conducted in Italy found that around half of vegan parents reported that their Primary Care Pediatrician (PCP) was unable to provide sufficient guidance on vegan weaning, while almost 80% stated that their PCP was against vegan weaning [13]. Similarly, 36.2% of parents who raise their children on a plant-based diet did not inform their primary care physician (PCP). In 70.8% of cases, the PCP was skeptical or opposed to the child’s fully plant-based diet. The most common professionals involved were dietitians, followed by medical dietitians [14].

Misinformation in this area can have serious consequences. Those who choose to follow a vegan diet by relying on their doctor may not receive the necessary knowledge about supplementation, monitoring, and proper planning of their diet. This can compromise their health and discredit the validity of a plant-based diet. To avoid this risk, VegPlate was created in 2017. VegPlate is a vegetarian food guide based on the Mediterranean diet that follows the established Italian Dietary Reference Intakes (DRIs). Its main objective is to help health professionals and individuals following a vegetarian or vegan diet to conveniently organize well-balanced meal plans [15].

Currently, there is a lack of knowledge regarding the health and supplementation behavior of Italian vegans. The study aimed to investigate the habits and behaviors of the Italian vegan population, including their vitamin and mineral supplementation practices, with a special focus on vitamin B12. Additionally, the study examined the frequency of consumption of processed foods and adherence to the Mediterranean food pyramid. The reasons behind the participants’ adoption of a vegan diet were also explored, as well as the main difficulties they faced in social settings and interactions with health professionals. The study aimed to assess possible critical issues. For research purposes, it was important to investigate the relationship between the participants’ social and dietary choices and their health status.

The study presents a qualitative analysis of lifestyle and perceived difficulties, as well as a quantitative analysis of supplements and staple foods based on the VegPlate recommendations [16].

## 2. Materials and Methods

### 2.1. Study Design and Participants

The study was conducted from 9 March to 6 April 2022 through a questionnaire. The questionnaire consisted of 46 items divided into 11 sections and was created using Google Forms. The total duration of the questionnaire varied between 8 and 12 min depending on individual responses. Each section explores different topics, including biographical and socio-economic information, motivation for choosing a vegan diet, eating habits and behavior, and questions on vitamin and mineral supplementation. The survey also includes questions on perceived difficulties in dealing with health professionals, as well as other general questions such as anthropometric data, personal choices, and types of vegan diets. The language used is clear, concise, and objective, with a formal register and precise word choice. The text adheres to conventional structure and formatting features, including consistent citation and footnote style. The grammar, spelling, and punctuation are correct. No changes in content have been made. The study employed structured and semi-structured questions, with some allowing for an “other” option to capture unexpected responses. For instance, participants were asked about unintentional exceptions, challenges in finding vegan options, and their interactions with nutrition professionals.

This study is a cross-sectional web-based survey that collects data on the dietary habits and lifestyle choices of the vegan population in Italy and abroad. The subjects were recruited primarily through social media channels, including Instagram, WhatsApp, Telegram, and Facebook. The sample was selected based on specific criteria:

- Italian nationality
- Adopting a vegan diet for more than 365 days
- Over 18 years of age

Prior to its use in this study, we pre-tested our survey for face validity with a sample of ten individuals who provided feedback on its comprehensibility, functionality, content, and completion time. Participation in the survey was voluntary and anonymous, and it could be terminated at any time without justification.

### 2.2. Data Assessment

The questionnaire was online for 1 month, from 9 March 2022 to 6 April 2022.

### 2.3. Data Analysis and Statistics

For each variable considered in the study, the absolute and percentage distributions of the subjects were calculated and supplemented where appropriate by indicators of centrality and variability. For each variable considered in the study, the absolute and percentage distributions of the subjects were calculated. For quantitative variables, the main indicators of centrality and variability were calculated.

Non-parametric tests were used to analyze comparisons between clinical variables in different groups. The normality of the distributions of the variables considered was checked prior to analysis. Numerical variables were analyzed using the Mann–Whitney U test and Kruskal–Wallis test, while categorical variables were analyzed using Pearson’s X2 test and Fisher’s exact test where appropriate. The correlation between ordinal variables was assessed using Spearman’s Rho correlation coefficient. Statistical significance was considered for *p*-values less than 0.05 (two-tailed test). IBM SPSS statistical software (version 20.0) was used for all analyses.

## 3. Results

### 3.1. Study Participants

The study included 2180 subjects who reported being vegan for more than one year (365 days). Of these, 69.1% (*n* = 1783) reported being vegan for between 1 and 5 years, 10.9% (*n* = 282) for between 5 and 10 years, and 4.5% (*n* = 115) for 10 years or more.

### 3.2. Sample Characteristics

The baseline characteristics of participants are shown in Table 1.

#### 3.2.1. Weight Status

The BMI of females and males differed by only one point, with females having a BMI of 21.7 kg/m^2^ and males having a BMI of 22.7 kg/m^2^. The percentage of underweight subjects (BMI < 18.5) was 10.2% for females and 7.1% for males, while the percentage of overweight subjects (BMI > 24.9) was 9.7% for females and 15.2% for males. Most participants in both groups had a normal weight (BMI 18.5–24.9), with 77.4% of females and 75% of males falling into this category. The rate of obesity (BMI > 30) was very low in both populations, with only 2.7% of females and 2.8% of males being classified as obese.

#### 3.2.2. Type of Vegan Diet

Of the participants, 95.6% (*n* = 2084) reported following a standard vegan diet, while 1.6% (*n* = 34) followed a high-protein vegan diet. A Whole-Food Plant-Based (WFPB) diet was followed by 0.4% (*n* = 8), a gluten-free vegan diet by 0.6% (*n* = 13), a low carbohydrate diet by 0.8% (*n* = 17), a raw vegan diet by 0.4% (*n* = 9), and a macrobiotic vegan diet by 0.7% (*n* = 15). None of the respondents followed strict fruitarian or liquid/juice-based vegan diets.

#### 3.2.3. Medical Conditions

Out of the 1805 subjects, 83.3% reported not having any chronic pathology. The remaining 16.7% reported suffering from various pathologies, including endometriosis (1.0%), asthma (3.7%), dyslipidemia (1.2%), chronic inflammatory bowel disease (1.5%), thyroiditis (3.3%), and other unidentified pathologies (5.1%).

### 3.3. Vegan Choice: MAIN Motivation

The main reasons for choosing a vegan diet are listed in Table 2.

**Figure 1:**
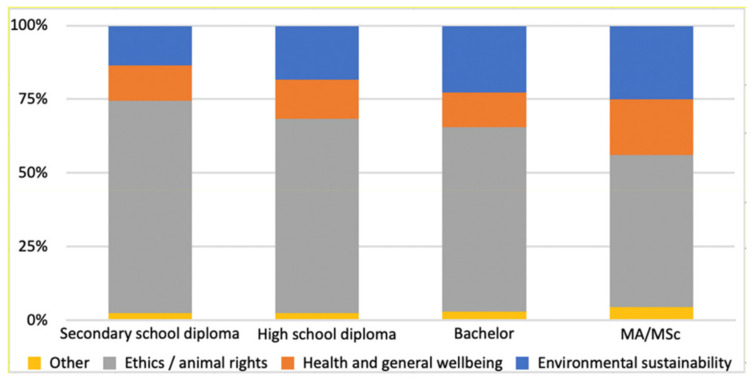
Correlation between higher educational qualification and the MAIN reason for the adoption of the vegan diet. Nominal variables were compared using the chi-square test.

A significant association (*p* = 0.005) was found between the level of higher education attained and the importance placed on environmental sustainability and health and wellbeing when adopting a vegan diet (Figure 1). However, no significant association was found for the other aspects analyzed, including ethics and animal rights, religious and spiritual beliefs, curiosity, and current trends.

**Figure 2:**
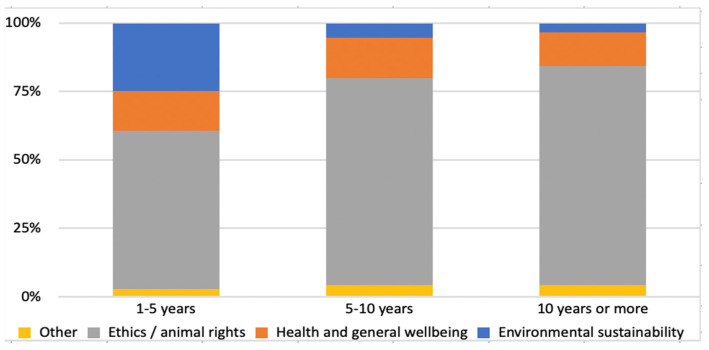
Correlation between the time of adoption of the vegan diet and MAIN reason for adopting the vegan diet. Nominal variables were compared using a chi-square test.

A negative significant association (*p* = 0.005) was found between the time of adopting a vegan diet and the importance of switching to such a diet for environmental sustainability reasons (Figure 2). Additionally, a positive significant association (*p* = 0.005) was found between the time of adopting a vegan diet and ethical motivations.

### 3.4. Vegan Diet in the Family

Of the subjects surveyed, 57.4% (*n* = 1365) claimed to be the only member of their family following a vegan diet. A total of 21.7% (*n* = 517) reported that their partner also follows this diet. A total of 8.7% (*n* = 207) declared that other family members are also vegan, 5.9% (*n* = 140) specified that either their father or mother is vegan, and 5.4% (*n* = 128) said at least one of their children is vegan. The remaining 0.9% (*n* = 22) reported that both parents are vegan.

#### 3.4.1. Children

Out of the 2180 participants, 88.6% (*n* = 1937) reported not having any children between the ages of 0 and 18 years. A total of 6.9% (*n* = 151) reported having one child, and 4.4% (*n* = 97) reported having more than one child.

#### 3.4.2. Weaning

Of the 356 children surveyed, 85 (23.9%) were raised with a vegan weaning, 49 (12.8%) with a vegetarian weaning, and 222 (63.3%) with an omnivorous weaning. A total of 37 (14.9%) of the subjects reported that their pediatrician supported vegan weaning, while an equal percentage, 37 (14.9%), reported that their pediatrician was against it. Of the respondents, 14.1% (*n* = 35) disagreed with the pediatrician but still weaned their child vegan. Meanwhile, 21.1% (*n* = 60) did not confront their pediatrician, and 32.1% (*n* = 80) had no intention of vegan weaning. Currently, 33.1% (*n* = 118) of the children follow an omnivorous diet, 26.7% (*n* = 95) follow a vegan diet, 14% (*n* = 50) follow a vegetarian diet, and 26.1% (*n* = 93) eat vegan at home but consume non-vegan food when outside. Of the children who are not currently following a vegan diet, 45.2% (*n* = 100) do not consider it a suitable choice at present, while 22.2% (*n* = 49) cite their partner’s opposition as the reason. A total of 17.2% (*n* = 38) state that their parent does not consider it a suitable choice, and 14.5% (*n* = 32) report that a vegan menu is not available in the canteen. Only 0.9% (*n* = 2) of cases cite opposition from the pediatrician as the reason.

#### 3.4.3. Exceptions

In the past year, 48.6% (*n* = 1060) of the participants reported no intentional deviations from their vegan diet. In 21.5% (*n* = 468) of cases, there were less than three deviations, while in 12.1% (*n* = 263) there were between three and five deviations. In 17.8% (*n* = 389) of cases, there were more than five deviations. Of the exceptions that occurred, 40.4% (*n* = 668) were due to a lack of other vegan options, 18% (*n* = 298) were to avoid disappointing others, 17.9% (*n* = 297) were due to a desire to taste, 14% (*n* = 231) were for other reasons, and 9.7% (*n* = 161) were to avoid feeling embarrassed. Overall, 96.1% (*n* = 2096) of respondents reported difficulty finding vegan options when dining out.

### 3.5. Frequencies

A significant correlation was found between the frequency of food consumption and the time of adoption of the vegan diet for some items (Table 3). Specifically, there was a negative correlation for the consumption of grains (ρ = −0.140, *p* < 0.001), legumes (ρ = −0.110, *p* < 0.001), soy products (ρ = −0.068, *p* = 0.002), and sugary drinks (ρ = −0.077, *p* < 0.001). On the other hand, there was a positive correlation for burgers and meat substitutes (ρ = +0.102, *p* < 0.001), coconut oil-based vegan cheeses (ρ = +0.139, *p* < 0.001), and nut-based vegan cheeses (ρ = +0.145 *p* < 0.001).

In addition, when comparing the time of adoption of a vegan diet with the importance of factors that determine the choice of frozen foods, preserved convenience foods, high-protein products, and meal replacements, a significant inverse correlation was found for speed (ρ = −0.52, *p* = 0.014) and curiosity (ρ = −0.86, *p* < 0.001).

No significant differences were found between the frequency of consumption of the analyzed food groups and educational qualifications, except in subjects with higher university qualifications who consumed more fruit and vegetables (*p* = 0.001) and nut-based vegan cheeses (*p* = 0.018) and fewer packaged and industrial products (*p* < 0.001).

### 3.6. Lifestyle

#### 3.6.1. Physical Activity, Tobacco and Alcohol

Of the respondents, 75.4% (*n* = 1643) reported practicing regular physical activity several times a week for 1–2 h, while 24.6% (*n* = 537) reported doing so sporadically. Non-smokers accounted for 56.7% (*n* = 1237) of the respondents, while ex-smokers accounted for 20% (*n* = 435). Occasional smokers and those who smoke less than 20 cigarettes per day accounted for 13.1% (*n* = 285) and 7.8% (*n* = 170), respectively. Only 0.3% (*n* = 7) reported smoking more than 20 cigarettes per day. In terms of alcohol consumption, 16.7% (*n* = 636) of participants reported never drinking alcohol, 27.6% (*n* = 602) reported drinking it a maximum of once per month, 37.8% (*n* = 824) reported drinking it 2 to 4 times per month, 15.9% (*n* = 347) reported drinking it 2 to 4 times per week, and 2.0% (*n* = 44) reported drinking it 5 or more times per week. Additionally, 80.7% (*n* = 1466) of participants reported drinking less than 2 alcoholic units.

#### 3.6.2. Medical Aspects

The results indicate that 49.2% (*n* = 1073) of the respondents undergo blood exams at least once a year to monitor their vitamin-mineral status. A total of 19.3% (*n* = 420) do so once every two years, while 21.4% (*n* = 466) and 10.1% (*n* = 221) do not undergo regular exams or rarely do so, respectively.

### 3.7. Relationship with Professionals in Nutrition

Of the participants, 69% (*n* = 1547) did not receive professional nutritional advice. Among those who did, 17.2% (*n* = 385) received advice from a nutritionist, 7% (*n* =158) from a dietitian, 6.3% (*n* = 142) from a medical dietitian, and 0.4% (*n* = 10) from a non-professional figure not belonging to any of the above categories. Of the total participants (*n* = 1209), 51.3% reported experiencing reticence from their general practitioner, 12.6% from gynecologists (*n* = 297), 10% from nutritionists (*n* = 235), 6% from pediatricians (*n* = 141), 5.3% from dietitians (*n* = 126), 6.5% from medical dietitians, and 8.3% from other medical professionals.

### 3.8. Source of Nutritional Knowledge

Of the respondents, 28.8% (*n* = 1761) usually source information about the vegan diet from the Internet (Instagram, blogs, groups, etc.), while 23.4% (*n* = 1426) rely on scientific articles. Additionally, 20.7% (*n* = 1238) seek advice from nutritional practitioners, 18.8% read books and magazines on the topic, 4.2% (*n* = 256) obtain information from acquaintances and friends, and 3.6% (*n* = 221) attend courses and seminars. Only 0.4% (*n* = 22) report never searching for nutritional information.

## 4. Discussion

This study examines the eating habits and behavior of the Italian vegan population, with particular attention to food frequency consumption, vitamin-mineral supplementation (Table 4), and their relationship with medical professionals. Most of the sample has followed a vegan diet for one year, with less than 5% following it for longer. These data further confirm the increasing interest in plant-based diets in recent years in Italy, as already shown in the Eurispes Italy Report [4].

In this study, most vegans were female (90.2%) and young (median age of 28 years), with a normal weight status. Only a small percentage of the total population was found to be underweight or overweight.

The surveyed population did not show any interest in restrictive and potentially dangerous dietary patterns, such as fruitarian or raw diets. In fact, almost all participants followed a standard vegan diet with no particular dietary restrictions within the plant food groups.

Over 80% of the total subjects did not have chronic pathologies. Less than 5% of the subjects had diseases associated with incorrect lifestyles, such as heart disease, type 2 diabetes mellitus, IBD (Inflammatory Bowel Disease), dyslipidemia, and arterial hypertension. Although the low average age of the sample must be taken into consideration, these results could confirm two hypotheses. First, an entirely plant-based diet may have positive effects on individuals’ health. Second, as suggested by the literature, those who follow a plant-based diet not only make more careful and conscious food choices but also tend to have a healthier and more active lifestyle in general than the omnivorous population, taking care of their health in all respects, as shown in other studies [10,17]. Further investigation is required to confirm this hypothesis.

Among the studied population, over half of the subjects shifted towards a plant-based diet due to ethical and animal rights concerns, followed by environmental sustainability, and lastly, health and general wellbeing. This is consistent with previous studies conducted in Australia, America, Canada, Great Britain, and Holland [18,19,20,21].

The study found a statistically significant positive correlation between higher reported educational qualifications and motivation for veganism related to environmental sustainability and general health and welfare. Conversely, a statistically significant negative correlation was found between higher reported educational qualifications and ethical motivation regarding animal welfare. With a higher level of education, individuals may become more aware of the impact of their diet on animal welfare, environmental sustainability, and personal health.

The study found a statistically significant negative correlation between the time of adoption of a vegan diet and motivations related to environmental sustainability and a statistically significant positive correlation between the time of adoption of a vegan diet and ethical motivations. It is possible that this finding reflects the fact that the younger generation is more aware of the environmental impact of their diet.

Regarding weaning, most children follow an omnivorous diet rather than a vegan or vegetarian one. This is consistent with other research that has shown that pediatricians are generally opposed to vegan weaning, a position that is also supported by the position paper of the Italian Society of Preventive and Social Pediatrics [13,14]. However, it is worth noting that a significant proportion of the subjects had no intention of introducing a vegan diet to their child(ren), indicating that the pediatrician’s opposition may not always be a decisive factor.

Although there is ample literature supporting the validity of a vegan diet even during early human development, some professionals remain unconvinced, possibly due to a lack of academic resources on the subject [10]. To eliminate uncertainties regarding plant-based diets in childhood, it is necessary to implement and offer educational and training courses aimed at pediatricians.

Currently, most children who are not vegan do not consider this dietary model suitable for themselves at this stage of their lives. The remaining percentage of non-vegan students is due to opposition from one or both parents or the unavailability of a fully vegan option at school. To address this issue, there is a strong need to incentivize canteens and the government to provide a fully plant-based menu in schools. 

According to the survey, a significant challenge faced by those transitioning to a vegan diet is the absence of entirely vegan options during social events. This often leads to intentional deviations from the plant-based diet. Although half of the participants claimed to have never strayed from their vegan diet, the other half admitted to making exceptions to their diet for various reasons, such as the desire to try new foods, avoid disappointing others, or avoid embarrassment. All the motivations mentioned in this study are also reflected in another research [22] and are confirmed by other results of this study. According to most respondents, it is still quite or very difficult to find a vegan option when eating out. For this reason, more than three quarters of the population analyzed in the study almost always eat meals at home. Promoting the integration of plant-based options into the menus of restaurants, canteens, hospitals, bakeries, and fast-food outlets to listen to consumer demands is therefore becoming an increasingly pressing need. This can help not only normalize this dietary pattern but also more easily convey that the vegan choice is not restrictive nor limiting.

However, research suggests that cooking at home more frequently is associated with a higher score on the Healthy-Eating Index 2015 [23]. Additionally, the literature shows that the vegan population adheres more closely to the Mediterranean diet than the vegetarian and omnivorous populations, consuming significantly more fruit, vegetables, pulses, and dried oleaginous fruit [24]. The study’s sample confirms that most subjects consume fruit and vegetables at least once a day, while over 80% of respondents consume legumes at least once a day. The consumption of oilseeds and nuts, as well as extra virgin olive oil, also followed similar trends. Regarding all the aforementioned categories, the frequency is in line with the guidelines for cereals and cereal products [25].

As per the same guidelines [25], sugary soft drinks and fried foods, referred to as “indulgent” foods, should be consumed occasionally and limited to special events. This recommendation is followed by almost all subjects. A very similar trend is also evident for frozen and preserved convenience products, coconut oil-based vegan cheeses, and ready meals, again demonstrating a similarity with what is stated in the frequency of consumption indications.

A deviation from the trends described above can be observed in the frequency of consumption of the category “burgers and meat substitutes”: one fifth of the respondents consume these products three to five times a week. As these are processed foods, their consumption should be limited as much as possible. However, this figure shows that vegans tend to consume more ultra-processed foods than vegetarians and omnivores [26]. Despite this, it is important to note that a vegan diet is still entirely plant-derived, cholesterol-free, often lower in calories and contains a fair proportion of fiber. Compared to their animal counterparts, plant-based products have demonstrated several health benefits [27,28]. Therefore, it would be inaccurate to categorize the vegan diet as nutritionally poor or unbalanced.

However, despite low consumption, a significant inverse correlation was found between the time of adoption of the vegan diet and the frequency of consuming grains, legumes, soy products, and sugary soft drinks. Conversely, a direct correlation was observed with the consumption of burgers and meat substitutes, coconut oil-based vegan cheeses, and nut-based vegan cheeses. This finding contrasts with previous studies [26]. A possible explanation for this phenomenon may be attributed to the increased purchasing power and knowledge of the aforementioned products over time.

The study found a statistically significant negative correlation between the duration of adopting a vegan diet and the selection of frozen foods, preserved convenience foods, high-protein products, and meal replacements that are associated with quick preparation and curiosity. This statement suggests that individuals who are more familiar with a vegan diet may be less inclined towards novelty and experimentation when it comes to their food choices.

The popularity of these foods among consumers can be attributed to their taste, which is the primary factor influencing their purchase. Many vegans reject the production methods of certain products but still desire the pleasure and enjoyment of eating. This is why the market for plant-based alternatives is rapidly expanding [29].

It is worth noting that respondents with higher educational qualifications consume fruit, vegetables, and fermented nut-based vegan cheeses more frequently, while packaged products are more commonly consumed by those with lower educational qualifications. This may be because individuals with higher educational qualifications are more health-conscious.

Focusing on lifestyle, it is evident from numerous reports in the literature that regular physical activity leads to better general health status, including clinical-metabolic, psychological, and behavioral benefits [30]. Sedentary behavior and unhealthy eating habits, which are often associated [31], play a decisive role in the spread of serious pathological frameworks, defined as “diseases of wellbeing” [32]. Based on these data, it is evident that over three quarters of the sample engage in regular physical activity several times a week for an average duration of 1–2 h, which is in line with WHO recommendations [33]. Additionally, the results indicate positive trends in smoking and alcohol consumption, with most of the sample being non-smokers or ex-smokers, as well as teetotalers or occasional drinkers. Alcohol consumption is widely recognized as a significant risk factor for cancer development, alongside smoking, obesity, physical inactivity, and poor fruit and vegetable consumption [34]. It is also a risk factor for blood diseases. Smoking, on the other hand, is a major risk factor for a range of diseases, including cancer and cardiovascular diseases.

As numerous position papers from various institutions have emphasized, vitamin B12 supplementation is essential for a nutritionally balanced and healthy vegan diet [10]. B12 is crucial for regulating hemoglobin and DNA synthesis, as well as for the proper functioning of the central nervous system. It is worth noting that no plant-based food contains B12. A deficiency in this area may lead to a range of health issues, including depression, memory disorders up to dementia, spinal cord problems up to tetra-paresis, and neuropathies affecting the peripheral nervous system [35].

It is worth noting that almost 90% of the sample is aware of these indications and regularly supplements accordingly. However, it is important to note and monitor the small percentage of the investigated population that claims never to use the supplement. Although this is a very small share, the health risks are significant and cannot be ignored. Therefore, it is crucial to continue informing patients and instructing medical professionals to ensure they have taken this recommendation on board. However, supplementing with iron, omega-3, and multivitamins is not as crucial as vitamin D supplementation. Almost one third of the analyzed sample regularly supplements with vitamin D. The body’s vitamin D levels are dependent on sun exposure. In the absence of adequate sun exposure, it must be obtained through fortified plant foods or supplements. This recommendation applies to the general population, not just vegans, as vitamin D intake is not dependent on diet [36].

It is noteworthy that despite less than 70% of the sample seeking support and having direct experience with a nutrition professional for vegan eating, almost three quarters of the total encountered resistance or skepticism from medical professionals, including general practitioners (GPs) who were reported to be the most skeptical among all other nutrition professionals. On one hand, this could indicate a potential lack of expertise and knowledge regarding vegan diets. On the other hand, this higher frequency could be due to medical figures being the most frequently addressed.

The reasons why professionals do not recommend a vegan diet are repeatedly refuted by the literature, as reported above. There is a need to educate health professionals, including nutrition specialists, about the validity of 100% plant-based diets. These diets can be applied to every patient at every stage of life and may have a preventive role in the onset of certain diseases if correctly planned and integrated. This issue could be addressed by implementing academic lessons during university and training courses for all health-related medical professionals, as previously mentioned.

Almost all the participants showed a strong desire to learn about vegan nutrition and actively sought out information from various sources, including nutrition professionals, scientific articles, books, magazines, friends, acquaintances, and online platforms such as Instagram, blogs, and groups. It is important to note that Instagram plays a significant role in disseminating content and facilitating the conscious and effective sharing of messages.

The study presented valuable results that can serve as a benchmark and for monitoring purposes. Additionally, the data can be used as a reference for other studies aimed at collecting information on the same indicators in specific contexts, which can then be compared with national-level data.

### Limitations of the Study

The study has some limitations. First, the sample only includes Italian participants, so it cannot be considered representative of other nations’ populations.

Additionally, there may be biases present in the study, including selection bias, performance bias, and recall bias.

The study may be subject to selection bias as participants were recruited on a voluntary basis through social media platforms such as Instagram, Facebook, and Telegram. The sample is limited to users subscribed to these channels and accounts of those who participated in the study’s dissemination. Therefore, any Italian vegans who did not fit into the aforementioned categories were excluded from the survey.

The study may be affected by performance bias since participants were aware that they were taking part in a study analyzing the eating habits of the vegan population in Italy, which could have influenced their responses. Furthermore, conducting the questionnaire online may result in less control over the respondent when compared to an in-person questionnaire.

Under this type of administration, it is not possible to verify the accuracy of the answers provided or the identity of the questionnaire respondent. Recall bias, however, may result from the possible inaccuracy or incompleteness of memories retrieved from study participants, particularly regarding food consumption frequency, vitamin-mineral supplementation, and other past experiences investigated.

## 5. Conclusions

In conclusion, our results indicate an increasing number of people choosing the vegan option, with a greater involvement among females compared to males. The main reasons for choosing a completely plant-based diet are ethics and animal rights.

Eating meals primarily at home is linked to better diet quality. In fact, the surveyed vegan population demonstrated greater attention to their eating habits compared to the general omnivorous population. The consumption of fruit, vegetables, cereals, legumes, oleaginous nuts, and extra virgin olive oil fully complies with the guidelines’ recommendations. Additionally, the use of processed and sugary products does not exceed the maximum intake limits indicated. Most of the survey sample also respects proper supplementation of vitamin B12, demonstrating widespread knowledge of the medical and nutritional indications reported by institutions.

Those who adopt a vegan diet, whether out of desire or necessity, often increase their nutritional knowledge and indirectly improve various other aspects of their lifestyle. This increased awareness is likely due to the desire to avoid potential deficiencies. The respondents showed a lower percentage of smokers and drinkers and a higher number of individuals who engage in regular physical activity.

The skepticism encountered by many individuals when approaching medical and health professionals about a plant-based diet is unfounded. Hypothesized deficiencies or the occurrence of physical and nutritional problems derived from the vegan diet are concepts that have been disproven by the literature. It is necessary to overcome these misconceptions.

Based on the findings, it may be beneficial to introduce educational courses and seminars or implement nutrition education classes in medical and health-profession degree programs. These courses should be led by professionals with expertise in plant-based nutrition, such as dietitians and specialized nutritional biologists. The purpose of this is to raise awareness among health professionals about the feasibility and validity of an all-plant-based diet and to provide their patients with up-to-date and scientifically based information. The main objective of the proposed interventions is to inform and educate health professionals about plant-based diets and thus reduce the risk of nutritional deficiencies in patients.

## Figures and Tables

**Table 1:** Baseline characteristics.

Total Subjects, %	100.0% (2180)
Females, %	90.2%
Males, %	8.4%
Others ^1^, %	1.4%
Age, years (median ± SD ^2^, range)	28 ± 8.9
Height, cm (median ± SD ^2^, range)	165.0 ± 7.6 (145–203)
Weight, kg (median ± SD ^2^, range)	58.0 ± 10.7 (37–135)
Total weight status	*n* = 2180
BMI ^3^, kg/m^2^ (mean ± SD ^2^, range)	21.8 ± 3.4 (14.7–45.1)
Underweight, % (BMI ^3^ < 18.5)	10.0%
Normal weight, % (BMI ^3^ 18.5–24.9)	77.0%
Overweight, % (BMI ^3^ 25–29.9)	10.2%
Obesity, % (BMI ^3^ > 30)	2.9%
Geographical area	
North-West Italy, %	38.8%
North-East Italy, %	27.3%
Central Italy, %	16.0%
South Italy, %	7.9%
Italian islands, %	4.3%
Abroad, %	5.8%
Marital status	
Engaged, %	31.6%
Single, %	28.0%
Cohabit, %	21.7%
Married, %	15.0%
Divorced, %	1.5%
Widowed, %	0.1%
Polyamorous relationship, %	0.1%
Profession	
Full-time employee, %	32.9%
Student, %	31.9%
Freelance worker, %	13.5%
Part-time employee, %	10.0%
Unemployed, %	4.4%
Housewife, %	2.2%
Student worker, %	1.9%
Retired, %	0.4%
Education	
High school diploma, %	42.3%
Bachelor, %	24.2%
MA/MSc, %	27.8%
Secondary school diploma, %	3.8%
PhD, %	1.7%

^1^ Other: not declared, non-binary, agender, transgender. ^2^ SD (Standard Deviation). ^3^ BMI (Body Mass Index) based on self-reported indications of body weight and size and calculated kg/m^2^.

**Table 2:** What is the MAIN reason that made you turn vegan?

	Percentage (%)
Total	100.0% (2180)
Environmental sustainability	21.2%
Health and general wellbeing	14.5%
Health (suffering from a chronic illness)	1.3%
Ethics/animal rights	61.2%
Food preferences	0.8%
Foods scandals	0.01%
Social influence (from friends, relatives, partner, etc.)	0.4%
Social justice/world’s hunger	0.5%
Curiosity/current trend	0.01%
Religious and spiritual beliefs	0.0%

**Table 3:** Frequency of different foods consumption.

Food	Frequency % (Total Population *n* = 2180)						
	>1/Day	1/Day	3–5/Week	1/Week	1 Every 2 Weeks	1/Month	Never
Fruit and vegetables	89.2%	8.1%	2.1%	0.5%	0.1%	0.0%	0.01%
Cereals (pasta, rice, bread…)	75.9%	19.7%	3.7%	0.5%	0.1%	0.0%	0.2%
Legumes (beans, lentils…)	42.9%	41.0%	14.5%	1.0%	0.2%	0.2%	0.1%
Soy products (tofu, tempeh…)	22.3%	37.8%	30.0%	6.6%	1.7%	0.8%	0.7%
Seeds and dried fruit	25.0%	43.4%	21.0%	6.3%	2.2%	1.4%	0.6%
Extra virgin olive oil	74.2%	20.2%	3.1%	1.0%	0.3%	0.4%	0.7%
Sugary drinks (Coca-Cola, Sprite…)	0.6%	0.6%	3.1%	14.6%	9.9%	20.3%	51.0%
Other vegetable oils (corn, peanut…)	3.6%	9.2%	21.2%	27.2%	12.0%	15.9%	10.9%
Industrial and packaged products (cookies, chips)	3.1%	16.8%	24.1%	27.3%	9.3%	12.9%	6.5%
Fried foods	0.0%	0.3%	3.4%	19.2%	22.6%	39.0%	15.4%
Meat alternatives (meatballs, burgers…)	0.5%	2.7%	20.4%	40.9%	14.3%	13.1%	8.3%
Coconut oil-based cheese substitutes	0.2%	0.5%	6.1%	15.0%	15.3%	27.0%	35.9%
Nuts-based cheeses	0.2%	0.8%	4.7%	11.9%	12.7%	29.1%	40.6%
Frozen ready meals (pizza, lasagna…)	0.01%	0.4%	2.4%	12.9%	10.1%	23.5%	50.5%
Preserved ready products (hummus, noodles…)	0.01%	0.7%	5.9%	10.9%	11.6%	20.7%	51.1%
High-protein products (bars, protein powder)	0.5%	3.2%	5.2%	4.6%	4.5%	9.3%	72.7%
Meal replacements	0.2%	0.1%	0.4%	0.7%	0.7%	1.7%	96.2%

**Table 4:** Vitamin-mineral supplementation.

Supplement	Regularly %	Occasionally %	By Prescription %	Never %
Vitamin B12	89.0%	6.3%	0.9%	3.7%
Vitamin D	28.9%	16.8%	9.3%	45.0%
Omega 3 (EPA ^1^/DHA ^2^)	8.2%	11.1%	1.7%	79.1%
Iron	4.2%	7.6%	7.3%	80.9%
Calcium	1.8%	4.0%	1.3%	92.9%
Multivitamin	6.0%	18.1%	2.8%	73.1%
Protein powder/BCAA ^3^/EAA ^4^	5.7%	9.0%	0.6%	84.7%
Other	5.6%	4.4%	1.8%	88.1%

^1^ EPA (Eicosapentenoic Acid). ^2^ DHA (Docosahexaenoic Acid). ^3^ BCAA (Branched-Chain Amino Acids). ^4^ EAA (Essential Amino Acids).

## Data Availability

The data presented in this study are available on request from the corresponding author.
